# The genome sequence of a caddisfly, *Limnephilus lunatus* (Curtis, 1834)

**DOI:** 10.12688/wellcomeopenres.18752.1

**Published:** 2023-01-17

**Authors:** Michael Austin, Caleala Clifford, Graham Rutt, Benjamin W. Price, Natural History Museum Genome Acquisition Lab, Wellcome Sanger Institute Tree of Life programme, Wellcome Sanger Institute Scientific Operations: DNA Pipelines collective, Tree of Life Core Informatics collective, Ian Wallace, Darwin Tree of Life Consortium

**Affiliations:** 1 Environment Agency, London, UK; 2 Natural Resources Wales, Cardiff, UK; 3 Life Science Department, Natural History Museum, London, UK; 4 British Caddis Recording Scheme, Wirral, UK

**Keywords:** Limnephilus lunatus, caddisfly, genome sequence, chromosomal, Trichoptera

## Abstract

We present a genome assembly from an individual *Limnephilus lunatus *(a caddisfly; Arthropoda; Insecta; Trichoptera; Limnephilidae). The genome sequence is 1,270 megabases in span. Most of the assembly is scaffolded into 13 chromosomal pseudomolecules, including the assembled Z chromosome. The mitochondrial genome has also been assembled and is 15.4 kilobases long.

## Species taxonomy

Eukaryota; Metazoa; Ecdysozoa; Arthropoda; Hexapoda; Insecta; Pterygota; Neoptera; Endopterygota; Trichoptera; Integripalpia; Plenitentoria; Limnephiloidea; Limnephilidae; Limnephilinae; Limnephilini; *Limnephilus*; *Limnephilus lunatus* Curtis, 1834 (NCBI:txid1218281).

## Background

**Figure 1.**
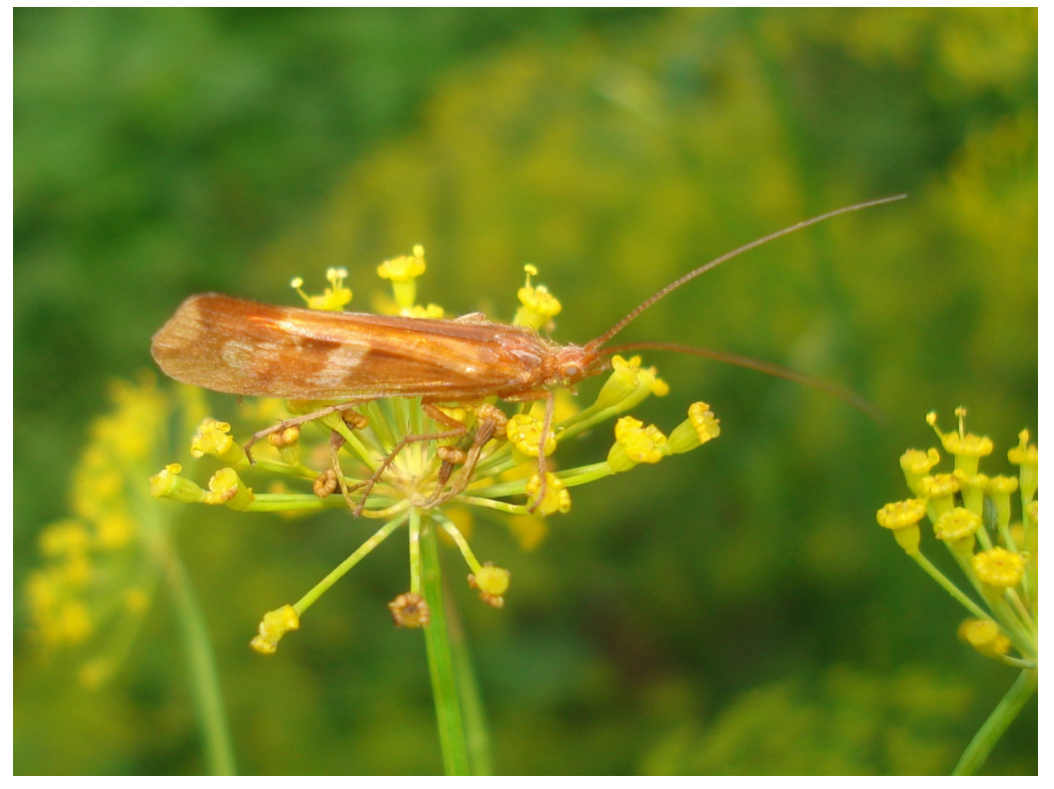
Photograph of *Limnephilus lunatus* by Sanja565658 (CC-SA).

*Limnephilus lunatus* ( Figure 1) is one of the most common British caddisflies, found from south-west England to Shetland and is one of the caddis that share the common name of ‘cinnamon sedge’. Larvae can be found in still and flowing waters of all sizes, but they are almost all permanent and do not dry up over summer. Larvae are found amongst submerged vegetation or debris such as twigs. The life cycle can vary but eggs are laid in late summer, with the egg mass being laid close to the waterline, with the larvae hatching if the egg mass is wetted or eventually by liquefaction of the jelly. Unusual populations from waters that dry up over summer are also laid in late summer and, if the larvae hatch early, they burrow into the substratum awaiting return of the water to start active growth. While adults from temporary waters have to emerge early before the site dries up, adults from permanent waters emerge at different times, then enter an ovarian diapause, but are otherwise active, until late summer and autumn.

The adult is quite variable with a pale semi-lunar mark at the wing termen being a consistent feature but one that is not unique to the species. It can be distinguished from its relatives using ( Barnard & Ross, 2012) and ( Wallace *et al.*, 2022). The larvae are of a group that makes their case by arranging the cut pieces of vegetation along the axis of the case; they have an irregular outline and are highly variable. The larvae can only be identified to species level when dead or anaesthetised using a key ( Wallace *et al.*, 2003), which only works for final and penultimate instars; there is no key to identify small larvae, pupae or eggs.

The high-quality genome sequence described here is, to our knowledge, the second reported for *L. lunatus*, with the first completed as part of the i5k initiative (assembly accession GCA_000648945.2). However, it is the first *L. lunatus* genome with chromosome-scale assembly, and has been generated as part of the Darwin Tree of Life project. It will aid in understanding the biology, physiology and ecology of the species, in addition to providing a mechanism to distinguish egg masses and early larval instars from those of its relatives.

## Genome sequence report

The genome was sequenced from one female *L. lunatus* specimen, collected from Tewin Bury Farm, UK (latitude 51.81, longitude –0.16). A total of 36-fold coverage in Pacific Biosciences single-molecule HiFi long reads and 44-fold coverage in 10X Genomics read clouds were generated. Primary assembly contigs were scaffolded with chromosome conformation Hi-C data. Manual assembly curation corrected 59 missing/misjoins and removed 7 haplotypic duplications, reducing the assembly length by 0.96% and the scaffold number by 47.95%.

**Figure 2.**
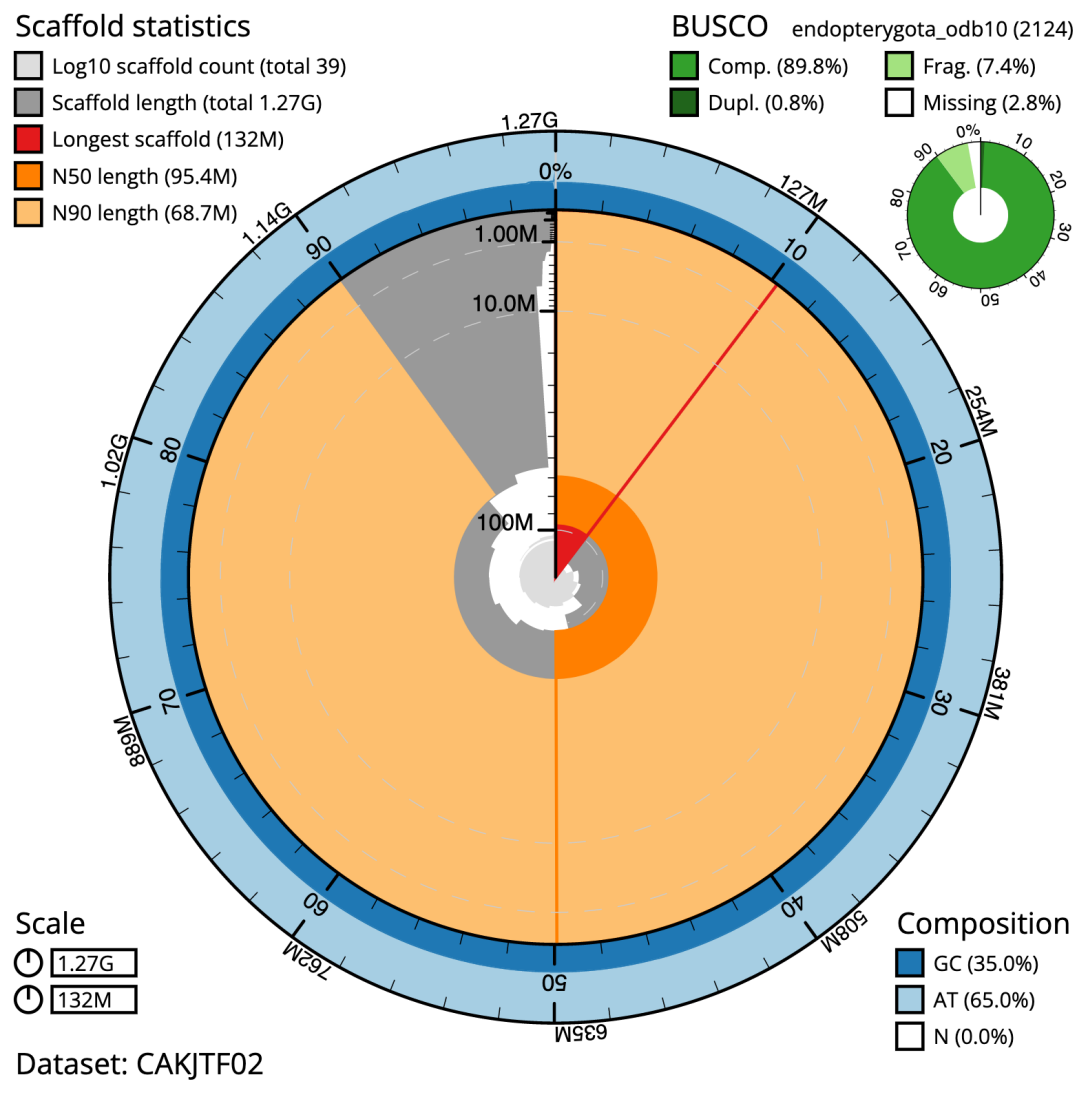
Genome assembly of *Limnephilus lunatus*, iiLimLuna2.2: metrics. The BlobToolKit Snailplot shows N50 metrics and BUSCO gene completeness. The main plot is divided into 1,000 size-ordered bins around the circumference with each bin representing 0.1% of the 1,269,651,477 bp assembly. The distribution of scaffold lengths is shown in dark grey with the plot radius scaled to the longest scaffold present in the assembly (131,514,635 bp, shown in red). Orange and pale-orange arcs show the N50 and N90 scaffold lengths (95,392,806 and 68,714,448 bp), respectively. The pale grey spiral shows the cumulative scaffold count on a log scale with white scale lines showing successive orders of magnitude. The blue and pale-blue area around the outside of the plot shows the distribution of GC, AT and N percentages in the same bins as the inner plot. A summary of complete, fragmented, duplicated and missing BUSCO genes in the endopterygota_odb10 set is shown in the top right. An interactive version of this figure is available at https://blobtoolkit.genomehubs.org/view/iiLimLuna2.2/dataset/CAKJTF02/snail.

The final assembly has a total length of 1,270 Mb in 38 sequence scaffolds with a scaffold N50 of 95.4 Mb ( Table 1). Most (98.84%) of the assembly sequence was assigned to 13 chromosomal-level scaffolds, representing 12 autosomes and the Z sex chromosome ( Figure 2– Figure 5; Table 2). Chromosome-scale scaffolds confirmed by the Hi-C data are named in order of size. Inversions between haplotypes were observed on chromosome 3 (104.64–110.84 Mb) and chromosome 11 (2.56–20.55 Mb). While not fully phased, the assembly deposited is of one haplotype. Contigs corresponding to the second haplotype have also been deposited.

**Table 1.** Genome data for *Limnephilus lunatus*, iiLimLuna2.2.

Project accession data		
Assembly identifier	iiLimLuna2.2	
Species	*Limnephilus lunatus*	
Specimen	iiLimLuna2	
NCBI taxonomy ID	1218281	
BioProject	PRJEB46311	
BioSample ID	SAMEA7521206	
Isolate information	iiLimLuna2 (genome assembly), iiLimLuna1 (RNA) iiLimLuna7 (Hi-C)	
Assembly metrics *		
Base pair QV	56.9	*(Benchmark: ≥ 50)*
*k*-mer completeness	99.99%	*(Benchmark: ≥ 95%)*
BUSCO **	C:89.8%[S:88.9%,D:0.8%],F:7.4%,M:2.8%,n:2,124	*(Benchmark: C ≥ 95%)*
Percentage of assembly mapped to chromosomes	98.84%	*(Benchmark: ≥95%)*
Sex chromosomes	Z	*(Benchmark: localised * *homologous pairs)*
Organelles	mitochondrial genome 15.4 kb	*(Benchmark: complete * *single alleles)*
Raw data accessions		
PacificBiosciences SEQUEL II	ERR6907853, ERR6939235	
10X Genomics Illumina	ERR6688476–ERR6688484	
Hi-C Illumina	ERR6688475	
PolyA RNA-Seq Illumina	ERR6688480	
Genome assembly		
Assembly accession	GCA_917563855.2	
*Accession of alternate * *haplotype*	GCA_917563845.2	
Span (Mb)	1269.6	
Number of contigs	138	
Contig N50 length (Mb)	19.0	
Number of scaffolds	38	
Scaffold N50 length (Mb)	95.4	
Longest scaffold (Mb)	131.5	

* Assembly metric benchmarks are adapted from column VGP-2020 of “Table 1: Proposed standards and metrics for defining genome assembly quality” from ( Rhie *et al.*, 2021). ** BUSCO scores based on the endopterygota_odb10 BUSCO set using v5.3.2. C = complete [S = single copy, D = duplicated], F = fragmented, M = missing, n = number of orthologues in comparison. A full set of BUSCO scores is available at https://blobtoolkit.genomehubs.org/view/iiLimLuna2.2/dataset/CAKJTF02/busco.

**Figure 3.**
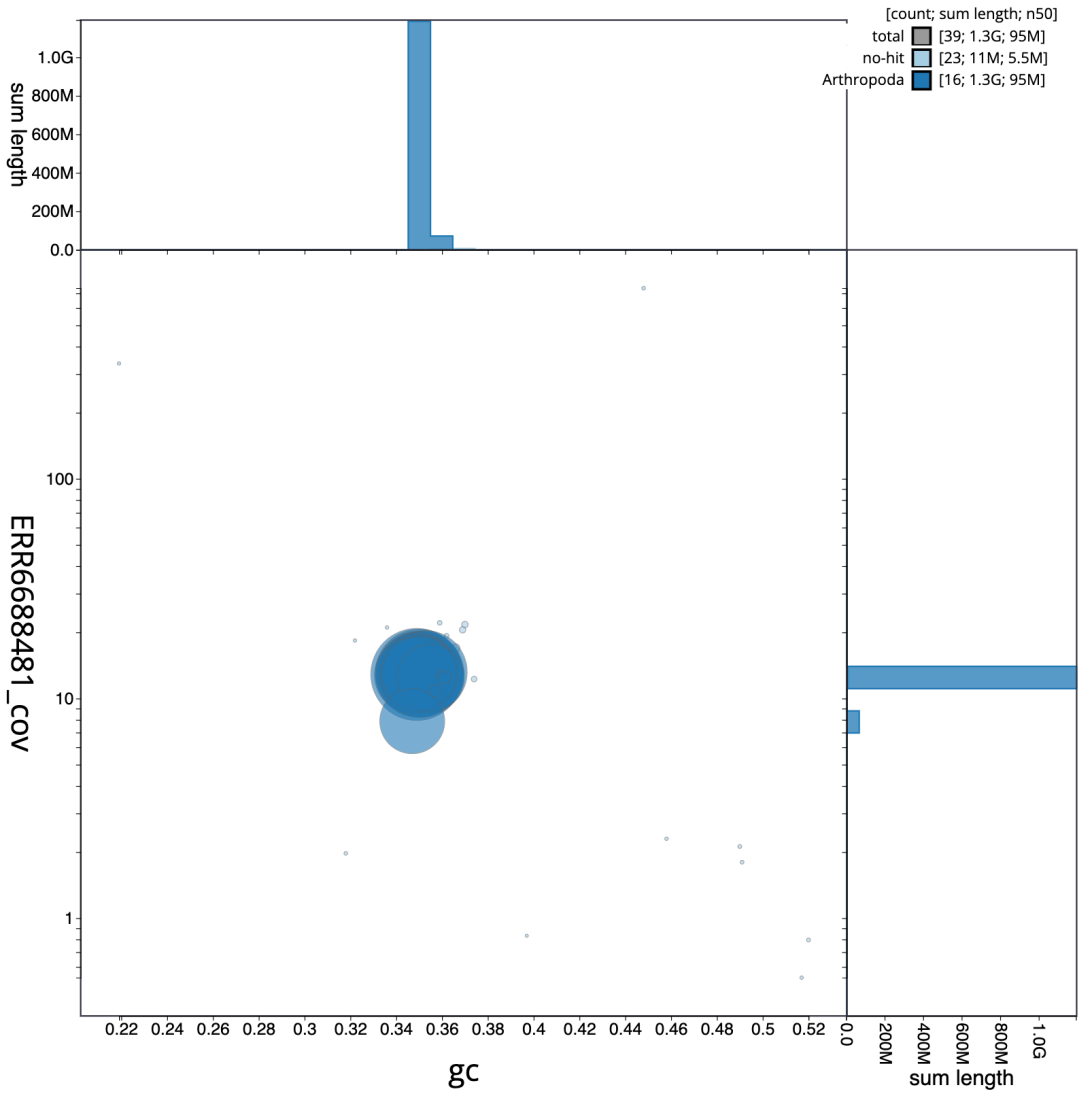
Genome assembly of *Limnephilus lunatus*, iiLimLuna2.2: GC coverage. BlobToolKit GC-coverage plot. Scaffolds are coloured by phylum. Circles are sized in proportion to scaffold length. Histograms show the distribution of scaffold length sum along each axis. An interactive version of this figure is available at https://blobtoolkit.genomehubs.org/view/iiLimLuna2.2/dataset/CAKJTF02/blob.

**Figure 4.**
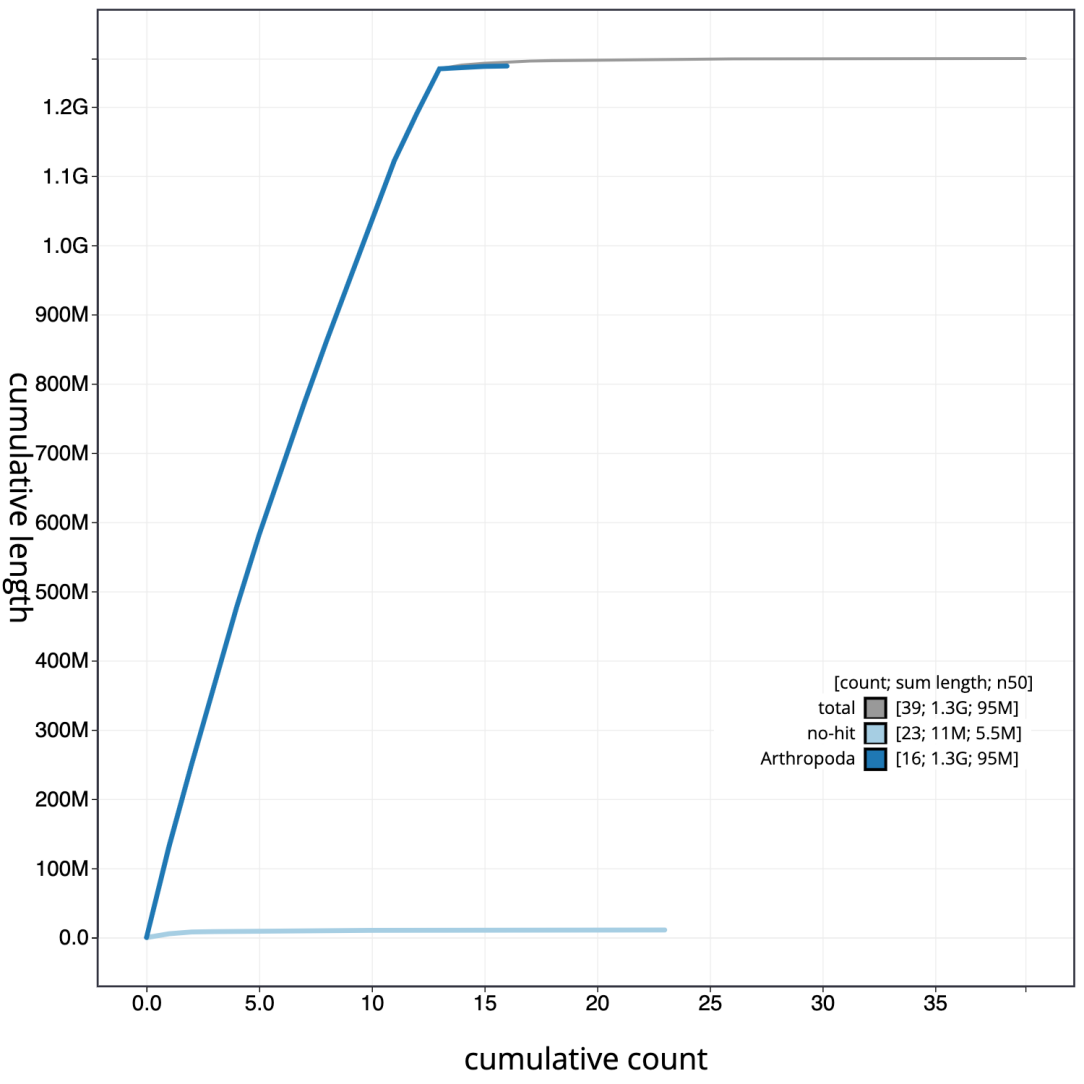
Genome assembly of *Limnephilus lunatus*, iiLimLuna2.2: cumulative sequence. BlobToolKit cumulative sequence plot. The grey line shows cumulative length for all scaffolds. Coloured lines show cumulative lengths of scaffolds assigned to each phylum using the buscogenes taxrule. An interactive version of this figure is available at https://blobtoolkit.genomehubs.org/view/iiLimLuna2.2/dataset/CAKJTF02/cumulative.

**Figure 5.**
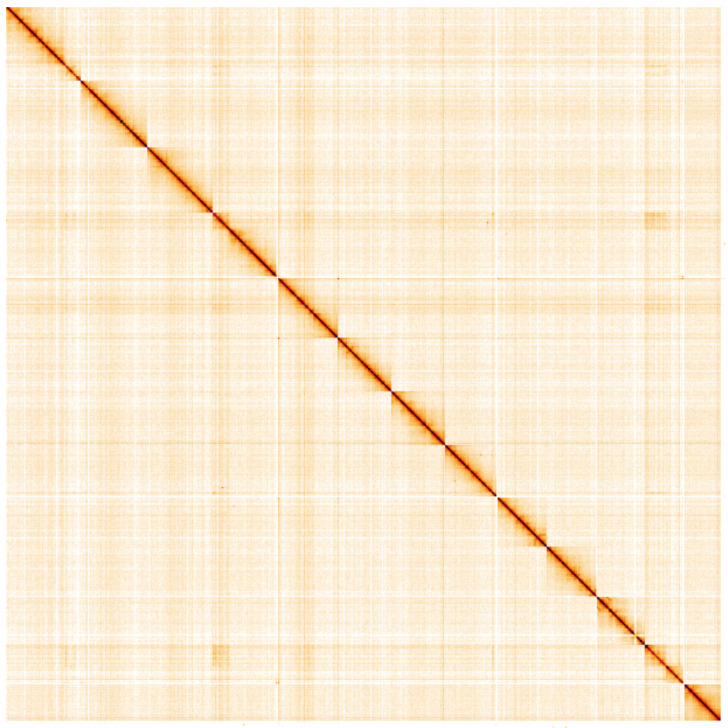
Genome assembly of *Limnephilus lunatus*, iiLimLuna2.2: Hi-C contact map. Hi-C contact map of the iiLimLuna2.2 assembly, visualised using HiGlass. Chromosomes are shown in order of size from left to right and top to bottom. An interactive version of this figure may be viewed at https://genome-note-higlass.tol.sanger.ac.uk/l/?d=BsyH8oUaSyWt3FzD7wFc3g.

The assembly has a BUSCO v5.3.2 ( Manni *et al.*, 2021) completeness of 89.8% using the OrthoDB-v10 endopterygota reference set. Although BUSCO coverage falls below the benchmark of 95%, the assembly is validated by high *k*-mer coverage and consensus quality QV scores ( Table 1).

**Table 2.** Chromosomal pseudomolecules in the genome assembly of *Limnephilus lunatus*, iiLimLuna2.

INSDC accession	Chromosome	Size (Mb)	GC%
OU830592.1	1	131.51	34.9
OU830593.1	2	118.19	35
OU830594.1	3	114.75	35
OU830595.1	4	112.84	35.1
OU830596.1	5	104.68	35.3
OU830597.1	6	95.39	35
OU830598.1	7	94.57	34.9
OU830599.1	8	90.41	35
OU830600.1	9	87.63	35
OU830601.1	10	87.37	34.7
OU830602.1	11	84.8	35
OU830603.1	12	68.71	35.5
OU830604.1	Z	64.07	34.7
OU830605.1	MT	0.02	21.9
-	unplaced	14.7	36.4

## Methods

### Sample acquisition and nucleic acid extraction

The *L. lunatus* specimen used for the genome assembly (iiLimLuna2) was collected and identified by Michael Austin (Environment Agency) from Tewin Bury Farm, Hertfordshire, UK (latitude 51.81, longitude –0.16). A second specimen (iiLimLuna7), which was used for RNA sequencing, was collected in Broadway Reen, Cardiff, Wales, UK (latitude 51.55, longitude –3.02) by Caleala Clifford (Natural Resources Wales). The specimen used for Hi-C analysis (iiLimLuna1) was collected from a pond in Gelli-hir Woods, Swansea, Wales, UK (latitude 51.62, longitude –4.08) by Graham Rutt (Natural Resources Wales). All specimens were collected from freshwater habitats using a kick-net. The specimens were preserved by snap-freezing in a dry shipper by Ben Price (Natural History Museum London).

DNA was extracted at the Tree of Life laboratory, Wellcome Sanger Institute. The iiLimLuna2 sample was weighed and dissected on dry ice with tissue set aside for Hi-C sequencing. Whole body tissue was cryogenically disrupted to a fine powder using a Covaris cryoPREP Automated Dry Pulveriser, receiving multiple impacts. High molecular weight (HMW) DNA was extracted using the Qiagen MagAttract HMW DNA extraction kit. Low molecular weight DNA was removed from a 20-ng aliquot of extracted DNA using 0.8X AMpure XP purification kit prior to 10X Chromium sequencing; a minimum of 50 ng DNA was submitted for 10X sequencing. HMW DNA was sheared into an average fragment size of 12–20 kb in a Megaruptor 3 system with speed setting 30. Sheared DNA was purified by solid-phase reversible immobilisation using AMPure PB beads with a 1.8X ratio of beads to sample to remove the shorter fragments and concentrate the DNA sample. The concentration of the sheared and purified DNA was assessed using a Nanodrop spectrophotometer and Qubit Fluorometer and Qubit dsDNA High Sensitivity Assay kit. Fragment size distribution was evaluated by running the sample on the FemtoPulse system.

RNA was extracted from whole body tissue of iiLimLuna7 in the Tree of Life Laboratory at the WSI using TRIzol, according to the manufacturer’s instructions. RNA was then eluted in 50 μl RNAse-free water and its concentration assessed using a Nanodrop spectrophotometer and Qubit Fluorometer using the Qubit RNA Broad-Range (BR) Assay kit. Analysis of the integrity of the RNA was done using Agilent RNA 6000 Pico Kit and Eukaryotic Total RNA assay.

### Sequencing

Pacific Biosciences HiFi circular consensus and 10X Genomics read cloud DNA sequencing libraries were constructed according to the manufacturers’ instructions. Poly(A) RNA-Seq libraries were constructed using the NEB Ultra II RNA Library Prep kit. DNA and RNA sequencing was performed by the Scientific Operations core at the WSI on Pacific Biosciences SEQUEL II (HiFi), Illumina HiSeq 4000 (RNA-Seq) and Illumina NovaSeq 6000 (10X) instruments. Hi-C data were also generated from iiLimLuna1 using the Arimav2 kit and sequenced on the Illumina NovaSeq 6000 instrument.

### Genome assembly

Assembly was carried out with Hifiasm ( Cheng *et al.*, 2021) and haplotypic duplication was identified and removed with purge_dups ( Guan *et al.*, 2020). One round of polishing was performed by aligning 10X Genomics read data to the assembly with Long Ranger ALIGN, calling variants with freebayes ( Garrison & Marth, 2012). The assembly was then scaffolded with Hi-C data ( Rao *et al.*, 2014) using SALSA2 ( Ghurye *et al.*, 2019). The assembly was checked for contamination as described previously ( Howe *et al.*, 2021). Manual curation was performed using HiGlass ( Kerpedjiev *et al.*, 2018) and Pretext ( Harry, 2022). The mitochondrial genome was assembled using MitoHiFi ( Uliano-Silva *et al.*, 2021), which performed annotation using MitoFinder ( Allio *et al.*, 2020). The genome was analysed and BUSCO scores generated within the BlobToolKit environment ( Challis *et al.*, 2020). Table 3 contains a list of all software tool versions used, where appropriate.

**Table 3.** Software tools and versions used.

Software tool	Version	Source
BlobToolKit	3.4.0	Challis *et al.*, 2020
freebayes	1.3.1-17- gaa2ace8	Garrison & Marth, 2012
Hifiasm	0.15.3	Cheng *et al.*, 2021
HIGlass	1.11.6	Kerpedjiev *et al.*, 2018
Long Ranger ALIGN	2.2.2	https://support.10xgenomics.com/ genome-exome/software/pipelines/ latest/advanced/other-pipelines
MitoHiFi	2.0	Uliano-Silva *et al.*, 2021
PretextView	0.2	Harry, 2022
purge_dups	1.2.3	Guan *et al.*, 2020
SALSA	2.2	Ghurye *et al.*, 2019

### Ethics/compliance issues

The materials that have contributed to this genome note have been supplied by a Darwin Tree of Life Partner. The submission of materials by a Darwin Tree of Life Partner is subject to the Darwin Tree of Life Project Sampling Code of Practice. By agreeing with and signing up to the Sampling Code of Practice, the Darwin Tree of Life Partner agrees they will meet the legal and ethical requirements and standards set out within this document in respect of all samples acquired for, and supplied to, the Darwin Tree of Life Project. Each transfer of samples is further undertaken according to a Research Collaboration Agreement or Material Transfer Agreement entered into by the Darwin Tree of Life Partner, Genome Research Limited (operating as the Wellcome Sanger Institute), and in some circumstances other Darwin Tree of Life collaborators.

## Data Availability

European Nucleotide Archive: *Limnephilus lunatus*, Accession number PRJEB46311; https://identifiers.org/ena.embl:PRJEB46311 ( Wellcome Sanger Institute, 2022) The genome sequence is released openly for reuse. The *Limnephilus lunatus* genome sequencing initiative is part of the Darwin Tree of Life (DToL) project. All raw sequence data and the assembly have been deposited in INSDC databases. The genome will be annotated using available RNA-Seq data and presented through the Ensembl pipeline at the European Bioinformatics Institute. Raw data and assembly accession identifiers are reported in Table 1.

## Author information

Members of the Natural History Museum Genome Acquisition Lab are listed here: https://doi.org/10.5281/zenodo.4790043.

Members of the Wellcome Sanger Institute Tree of Life programme are listed here: https://doi.org/10.5281/zenodo.4783585.

Members of Wellcome Sanger Institute Scientific Operations: DNA Pipelines collective are listed here: https://doi.org/10.5281/zenodo.4790455.

Members of the Tree of Life Core Informatics collective are listed here: https://doi.org/10.5281/zenodo.5013541.

Members of the Darwin Tree of Life Consortium are listed here: https://doi.org/10.5281/zenodo.4783558.
