# Supplementary material for: The genome sequence of a caddisfly, Limnephilus lunatus (Curtis, 1834)
Source: Wellcome Open Res. 2023 Jan 17;8:25. [Version 1] doi: 10.12688/wellcomeopenres.18752.1 (PMC10318372; doi:10.12688/wellcomeopenres.18752.1)
Supplement: Peer review history

# Peer review: Reviewer response for version 1

**DOI:** 10.21956/wellcomeopenres.20792.r61662

**Published:** 2023-07-03

**Authors:** Darren J Obbard

**Affiliations:** 1 The University of Edinburgh, Edinburgh, Scotland, UK

The authors present a high-quality genome for the caddisfly, *Limnephilus lunatus*. The methods are state-of-the-art and are fully described. I have no concerns about the research, and my comments are minor – pertaining only to the content and writing of this genome note. 

1. I found the background section hard to read: the sentences do not seem to be ordered in a clear narrative, and the grammar is poor (missing commas, changing subjects). I think adding 50% words and explaining things more clearly / fully would be a great help. E.g.:
  “ *Larvae can be found in still and flowing waters of all sizes, but they are almost all permanent and do not dry up over summer*”. The larvae are permanent and do not dry up? I assume not, but the larvae are the subject of this sentence. 
  “ *… with the larvae hatching if the egg mass is wetted or eventually by liquefaction of the jelly*.” What is it that happens 'eventually'? Is this supposed to read “ *… with the larvae hatching if the egg mass is wetted, or eventually being released by liquefaction of the jelly.*”
   “ *The larvae are of a group that ..*” Surely the adults and large are in the same group? Perhaps “ *In this, and related, species the larvae …*” or “ *In this group of species, the larvae …*”
  “. *.they have an irregular outline and are highly variable*” The larvae or the cases? Again, the subject is the larvae, but seems to switch here to cases.
2. Also, “ *… and is one of the caddis …*“ I am not familiar with this use of caddis rather than caddisfly (the discussion refers to the species, not the larva) and I would expect “caddisflies”. It is also the only time this is used in the paper.
3. There are no citations to any non-taxonomic literature. It is a great shame this work is not embedded within the literature that has used caddisflies for bioassays or pollution studies. Also no mention is made of the potential utility of this sequence for evolutionary questions, for example in studies of sex chromosome evolution ^1^. 
4. I am surprised the i5K initiative paper that previously sequenced this species is not cited ^2^.
5. I presume the apparently poor Busco completeness is likely to reflect the Busco gene set rather than the assembly, It would be good to see some comment on this.

Are sufficient details of methods and materials provided to allow replication by others?

Yes

Is the rationale for creating the dataset(s) clearly described?

Partly

Are the datasets clearly presented in a useable and accessible format?

Yes

Are the protocols appropriate and is the work technically sound?

Yes

Reviewer Expertise:

Evolutionary genetics and comparative genomics of insects and invertebrate pathogens

I confirm that I have read this submission and believe that I have an appropriate level of expertise to confirm that it is of an acceptable scientific standard.

## References

[1] : Sex chromatin and sex chromosome systems in nonditrysian Lepidoptera (Insecta). *Journal of Zoological Systematics and Evolutionary Research* .2000;38(2) : 10.1046/j.1439-0469.2000.382130.x 73-79 10.1046/j.1439-0469.2000.382130.x

[2] : Gene content evolution in the arthropods. *Genome Biol* .2020;21(1) : 10.1186/s13059-019-1925-7 15 10.1186/s13059-019-1925-7 31969194PMC6977273

# Peer review: Reviewer response for version 1

**DOI:** 10.21956/wellcomeopenres.20792.r59929

**Published:** 2023-06-22

**Authors:** Monica Poelchau

**Affiliations:** 1 National Agricultural Library, Beltsville, Maryland, USA

This article describes the sequencing and assembly of the *Limnephilus lunatus* genome, and provides standard assembly quality metrics. The genome quality is a tremendous improvement over the previous genome assembly by the i5k pilot project, and should be a welcome resource for arthropod genomics research.

 My only suggestions are to:

1. Include citations for the assertions about the species' biology in the introduction;
2. When referring to the previous i5k assembly, cite the Thomas *et al.,* paper ^1^ that first presented and used the original assembly.

Are sufficient details of methods and materials provided to allow replication by others?

Yes

Is the rationale for creating the dataset(s) clearly described?

Yes

Are the datasets clearly presented in a useable and accessible format?

Yes

Are the protocols appropriate and is the work technically sound?

Yes

Reviewer Expertise:

Genomics, databases

I confirm that I have read this submission and believe that I have an appropriate level of expertise to confirm that it is of an acceptable scientific standard.

## References

[1] : Gene content evolution in the arthropods. *Genome Biol* .2020;21(1) : 10.1186/s13059-019-1925-7 15 10.1186/s13059-019-1925-7 31969194PMC6977273
